# GH Dysfunction in Engrailed-2 Knockout Mice, a Model for Autism Spectrum Disorders

**DOI:** 10.3389/fped.2014.00092

**Published:** 2014-09-01

**Authors:** Giovanni Provenzano, Elena Clementi, Sacha Genovesi, Manuela Scali, Prem Prakash Tripathi, Paola Sgadò, Yuri Bozzi

**Affiliations:** ^1^Laboratory of Molecular Neuropathology, Centre for Integrative Biology (CIBIO), University of Trento, Trento, Italy; ^2^Neuroscience Institute, National Research Council (CNR), Pisa, Italy; ^3^Laboratory of Neurobiology, Scuola Normale Superiore, Pisa, Italy

**Keywords:** autism spectrum disorders, hippocampus, pituitary gland, liver, growth hormone, insulin-like growth factor, neuroendocrine axis, mouse model

## Abstract

Insulin-like growth factor 1 (IGF-1) signaling promotes brain development and plasticity. Altered IGF-1 expression has been associated to autism spectrum disorders (ASD). IGF-1 levels were found increased in the blood and decreased in the cerebrospinal fluid of ASD children. Accordingly, IGF-1 treatment can rescue behavioral deficits in mouse models of ASD, and IGF-1 trials have been proposed for ASD children. IGF-1 is mainly synthesized in the liver, and its synthesis is dependent on growth hormone (GH) produced in the pituitary gland. GH also modulates cognitive functions, and altered levels of GH have been detected in ASD patients. Here, we analyzed the expression of GH, IGF-1, their receptors, and regulatory hormones in the neuroendocrine system of adult male mice lacking the homeobox transcription factor Engrailed-2 (*En2*^−/−^ mice). *En2*^−/−^ mice display ASD-like behaviors (social interactions, defective spatial learning, increased seizure susceptibility) accompanied by relevant neuropathological changes (loss of cerebellar and forebrain inhibitory neurons). Recent studies showed that *En2* modulates IGF-1 activity during postnatal cerebellar development. We found that GH mRNA expression was markedly deregulated throughout the neuroendocrine axis in *En2*^−/−^ mice, as compared to wild-type controls. In mutant mice, GH mRNA levels were significantly increased in the pituitary gland, blood, and liver, whereas decreased levels were detected in the hippocampus. These changes were paralleled by decreased levels of GH protein in the hippocampus but not other tissues of *En2*^−/−^ mice. IGF-1 mRNA was significantly up-regulated in the liver and down-regulated in the *En2*^−/−^ hippocampus, but no differences were detected in the levels of IGF-1 protein between the two genotypes. Our data strengthen the notion that altered GH levels in the hippocampus may be involved in learning disabilities associated to ASD.

## Introduction

Insulin-like growth factor 1 (IGF-1) is a hormone primarily produced by the liver, which exerts an endocrine action on multiple target tissues. It is also locally produced in tissues, including brain, where it acts in a paracrine/autocrine fashion. Several studies demonstrate that IGF-1 profoundly modulates brain function, both during development and adult life. During development, IGF-1 promotes neuronal survival [reviewed in Ref. (1)] and maturation of cortical and retinal function (2–4), whereas in adult life, it exerts multiple actions ranging from the control of synaptic plasticity to neuroprotection [reviewed in Ref. (1, 5)]. During adulthood, IGF-1 is crucial for both basal and exercise-induced hippocampal neurogenesis (6, 7) and markedly regulates learning and cognition (8).

A link between IGF-1 pathway and autism spectrum disorders (ASD) has been proposed. IGF-1 levels have been found increased in the blood (9) and decreased in the cerebrospinal fluid (10, 11) of ASD children, as compared to healthy individuals. In addition, deregulation of IGF-1 signaling pathway, which involves downstream effectors such as PI3 kinase (PI3K), protein kinase B (AKT), and mammalian target of rapamycin (mTOR), has been shown in syndromic forms of ASD [reviewed in Ref. (12)]. This led to propose that IGF-1 treatment might be beneficial for ASD, and clinical trials have been approved^1^ . Indeed, IGF-1 administration is able to rescue ASD-related molecular changes in neurons derived from ASD patients (13) as well as ASD-like behavioral deficits in mouse models (14, 15). Altered levels of other hormones of the IGF-1 pathway have been associated to ASD (9). IGF-1 synthesis in the liver depends on levels of circulating growth hormone (GH) produced in the pituitary gland. GH also modulates cognitive functions (16), and altered GH levels have been detected in ASD patients (17).

Genome-wide association studies identified the transcription factor Engrailed-2 (*En2*) as a candidate gene for ASD (18), and two recent studies confirmed that *En2* expression is altered in the cerebellum of ASD patients (19, 20). Mice lacking the homeobox domain of *En2* [*En2^hd/hd^* mice; (21); here referred to as *En2*^−/−^] are considered a suitable animal model to study the neurodevelopmental basis of ASD. *En2*^−/−^ mice display neuropathological and behavioral changes relevant to ASD. Reduced social interactions, defective spatial learning (22–24), and increased seizure susceptibility (25) accompanied by neuropathological changes relevant to ASD [loss of cerebellar and forebrain inhibitory neurons; (26, 27)] were indeed described in *En2*^−/−^ mice. In addition, we recently showed that several genes related to ASD are markedly deregulated in the cerebellum and hippocampus of *En2*^−/−^ mice (28), thus indicating that *En2* mutants are a reliable model to investigate gene expression changes relevant to ASD.

A recent study established an important link between *En2* and the modulation of IGF-1 pathway (29). During postnatal development, *En2* controls proliferation and differentiation of cerebellar granule neuron precursors (GNPs). Notably, in postnatal *En2*^−/−^ cerebellum, the activity of downstream effectors of IGF-1 is increased, and IGF-1 has a stronger mitogenic effect on GNPs, as compared to WT (29). These results indicate that *En2* negatively regulates IGF-1 signaling during postnatal cerebellar development.

Here, we investigated the relationship between *En2* and GH/IGF-1 pathway in the neuroendocrine system. We analyzed the expression of GH, IGF-1, their receptors, and regulatory hormones in the brain–pituitary–liver axis of adult wild-type (WT) and *En2*^−/−^ mice. We show that GH levels are lowered in the hippocampus of *En2*^−/−^ mice, suggesting that this alteration might contribute to learning disabilities in this ASD mouse model.

## Materials and Methods

### Animals

Experiments were conducted in conformity with the European Community Directive 2010/63/EU and were approved by the Italian Ministry of Health. Animals were housed in a 12-h light/dark cycle with food and water available *ad libitum*, and all efforts were made to minimize animal suffering during the experiments. The generation of *En2* mutants were originally generated on a mixed 129Sv × C57BL/6 genetic background (21) and then backcrossed at least five times into a C57BL/6 background (27). *En2*^+/+^ (WT) and *En2*^−/−^ mice used in this study were obtained by heterozygous mating (*En2*^±^ × *En2*^±^) and genotyped by PCR as previously described (27). A total of 34 adult (3–5 months old) male mice were used: 6 mice per genotype for quantitative RT-PCR, 5 mice per genotype for enzyme-linked immuno-sorbent assay (ELISA) tests, 3 mice per genotype for immunohistochemistry, and 3 mice per genotype for *in situ* hybridization.

### Quantitative RT-PCR

Tissues from WT and *En2*^−/−^ mice (*n* = 6 per genotype) were dissected and frozen in dry ice. Blood samples were centrifuged in an Eppendorf benchtop centrifuge for 15 min at 3,000 rpm to separate serum from cell fraction, and then frozen in dry ice. All samples were stored at −80°C until use. Total RNAs were extracted by Trizol reagent (Invitrogen), treated with DNAse, purified by the RNAeasy Kit (Qiagen), and pooled. cDNAs were synthesized from pooled RNAs (3 μg) by SuperScript VILO cDNA Synthesis Kit (Invitrogen). Quantitative reverse-transcription PCR (RT-PCR) was performed in a C1000 thermal cycler (BioRad) with real-time detection of fluorescence, using the KAPA SYBR FAST master mix reagent (Resnova). Mouse mitochondrial ribosomal protein L41 (Mrpl41) was used as a standard for quantification. Primers (Sigma Genosys, UK) sequences are reported in Table 1. Ratios of comparative concentrations of each mRNA with respect to L41 mRNA were then calculated and plotted as the average of three to four independent reactions with technical replicates obtained from each RNA pool. Expression analyses were performed using the CFX3 Manager (BioRad) software.

**Table 1:** **Primers used for quantitative RT-PCR experiments**.

Gene	GenBank no.	Forward primer (5’–3’)	Reverse primer (5’–3’)
En2	NM_010134.3	ACTGCACGCGCTATTCTG	ACCTGTTGGTCTGAAACTCAG
GH	NM_008117.3	GCAATGGCTACAGACTCT	AAACAGACTGGACAAGGG
GHR	NM_001286370.1	CGTTCCCCTGAACTGGAGAC	CAGCTTGTCGTTGGCTTTCC
IGF-1 class 1	NM_001111275.1	AGCGATGGGGAAAATCAGCA	CAGAGCGCCAGGTAGAAGAG
IGF-1 class 2	NM_001111276.1	CTGATGTCTGGTCCTTCGGG	CACCCTCCATGACGAAACGA
IGF-1R	NM_010513.2	CTGATGTCTGGTCCTTCGGG	CACCCTCCATGACGAAACGA
mGRF	NM_010285.2	AGGATCCAGGAACAAAGGGC	GCAAGATGCTCTCCAGGGTC
SST	NM_009215	AGGACGAGATGAGGCTGG	CAGGAGTTAAGGAAGAGATATGGG

*Abbreviations are as in the text*.

### Bioinformatic analysis

Analysis of *En2* binding sites on GH, GHR, mGRF, SST, IGF-1, and IGF-1R gene promoters was performed using the Matinspector web-based search algorithm available from Genomatix Software^2^ . The algorithm calculated the similarity between the core motif and overall sequence of the *En2* consensus binding site (matrix ID: V$EN2.01) and the gene promoter sequences of interest. The *En2* matrix was determined based on the Genomatix Matrix Library Version 9.1.

### Immunohistochemistry

Brains from adult WT and *En2*^−/−^ mice (*n* = 3 per genotype) were used for immunohistochemical characterization of hypothalamic SST. Brains were fixed by transcardial perfusion with 4% paraformaldehyde followed by 1 h post-fixation, and coronal sections (40 μm thickness) were cut by a vibratome (Leica). Serial sections at level of the hypothalamus were incubated overnight with and anti-SST rabbit polyclonal (Peninsula-Bachem; 1:2000 dilution). Signals were revealed using appropriate secondary antibodies and fluorophores as described (27). Hypothalamic nuclei were identified according to the Allen Brain Atlas^3^ . Images were acquired at 10× objective magnification using a Zeiss AxioImager M2 microscope.

### *In situ* hybridization

Brains from WT and *En2*^−/−^ mice (*n* = 3 per genotype) were rapidly removed and frozen on dry ice. Coronal cryostat sections (20 μm thick) were fixed in 4% paraformaldehyde. Non-radioactive *in situ* hybridization was performed as previously described (27) using digoxigenin-labeled riboprobes specific for GH [GenBank ID: X02891; (30)], mGRF (31), and IGF-1 (GenBank ID: NM_010512). The IGF-1 cDNA was cloned by PCR from hippocampal cDNA and recognizes both class 1 and class 2 IGF-1 mRNAs. Signal was detected by alkaline phosphatase-conjugated anti-digoxigenin antibody followed by alkaline phosphatase staining. The specificity of the results was confirmed by the use of sense riboprobes (not shown).

### Enzyme-linked immuno-sorbent assay

Blood samples (*n* = 5 per genotype) were collected at the time of animal sacrifice, kept in ice for 30 min, and then centrifuged in an Eppendorf benchtop centrifuge for 15 min at 3,000 rpm to separate serum. Dissected liver and hippocampal tissues (*n* = 5 per genotype) were homogenized in lysis buffer (50 mM Tris–HCl pH 7.5, 1% NP-40, 1% Triton-100, 1 mM PMSF, 10% glycerol, and protease inhibitor cocktail; Sigma-Aldrich). Homogenates were incubated in ice for 30 min, centrifuged at 12,000 rpm for 5 min at 4°C, and supernatants were recovered and stored at −80°C. Protein concentration in serum, liver, and hippocampal samples was determined by BCA method (Pierce). Samples were processed for GH (EZRMGH-45K, Millipore) and IGF-1 (ELM-IGFI; RayBiotech) ELISA according to manufacturers’ protocol. All samples were analyzed in duplicate.

### Statistical analysis

Statistical analysis was performed with SigmaPlot 11.0 and Prism 6 (GraphPad) softwares. Values were expressed as mean ± SEM and quantitative gene expression differences between WT and *En2*^−/−^ mice were assessed by Student’s *t*-test, with the level of statistical significance set at *p* < 0.05.

## Results

### Expression of *En2* and IGF-1 signaling genes in the mouse brain–pituitary–liver axis

**Figure 1:**
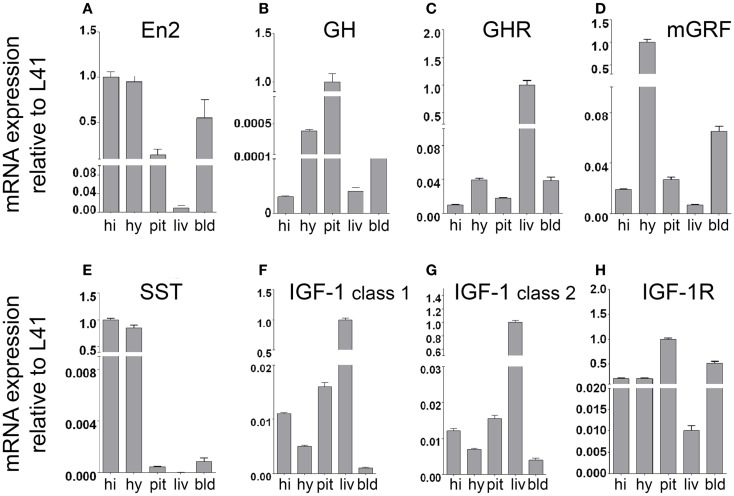
**mRNA expression of *En2* and genes involved in the GH/IGF-1 pathway in the neuroendocrine axis of WT mice**. *En2* **(A)**, GH **(B)**, GHR **(C)**, mGRF **(D)**, SST **(E)**, IGF-1 **(F,G)**, and IGF-1R **(H)** mRNA expression levels in the hippocampus, hypothalamus, pituitary gland, liver, and blood, obtained by quantitative RT-PCR. For each mRNA, relative expression levels (normalized to L41) are reported on a log scale. Two different transcripts (class 1 and class 2) were analyzed for IGF-1. Values are plotted as mean ± SEM of three independent experiments. Abbreviations: hi, hippocampus; hy, hypothalamus; pit, pituitary gland; liv, liver; bld, blood (cell fraction). Other abbreviations are as in the text.

By using quantitative RT-PCR, we first investigated mRNA expression of *En2*, GH, GH receptor (GHR), mGRF, SST, IGF-1, and IGF-1 receptor (IGF-1R) in the neuroendocrine axis of WT adult male mice from our colony. mRNA expression was studied in the hypothalamus, pituitary gland, and liver (the crucial tissues involved in GH and IGF-1 synthesis), as well as hippocampus and blood cell fraction. In agreement with previous findings, we confirmed that *En2* mRNA is expressed in the hypothalamus and hippocampus (23, 25, 27). *En2* mRNA was also expressed at detectable levels in blood, pituitary gland, and liver (Figure 1A). As expected, GH mRNA was mainly detected in the pituitary gland, while much lower levels were present in the hippocampus, hypothalamus, liver, and blood (Figure 1B). Consistent with the notion that liver is the main target of GH action, we found GHR mRNA predominantly expressed in the liver, and at lower levels in the other tissues analyzed (Figure 1C). mRNA expression of mGRF and SST, the two hypothalamic hormones controlling GH synthesis, was mainly detected in the hypothalamus (Figures 1D,E). High levels of SST mRNA were also present in the hippocampus, as previously described (27). Two major different transcripts have been described for IGF-1 [class 1 and class 2; (32, 33)]. Both IGF-1 class 1 and class 2 mRNAs were predominantly expressed in the liver (Figures 1F,G), as expected (32). Finally, IGF-1R mRNA was mainly expressed in the pituitary gland (the target for IGF-1 negative feedback for GH production) (Figure 1H), but also throughout the neuroendocrine axis, consistent with the widespread action of IGF-1 on multiple tissues. These results clearly indicate that our RT-PCR protocol can detect the expression of genes belonging to the GH/IGF-1 pathway in the appropriate tissues throughout the brain–pituitary–liver axis.

### Altered GH mRNA expression in the brain–pituitary–liver axis of *En2*^−/−^ mice

**Figure 2:**
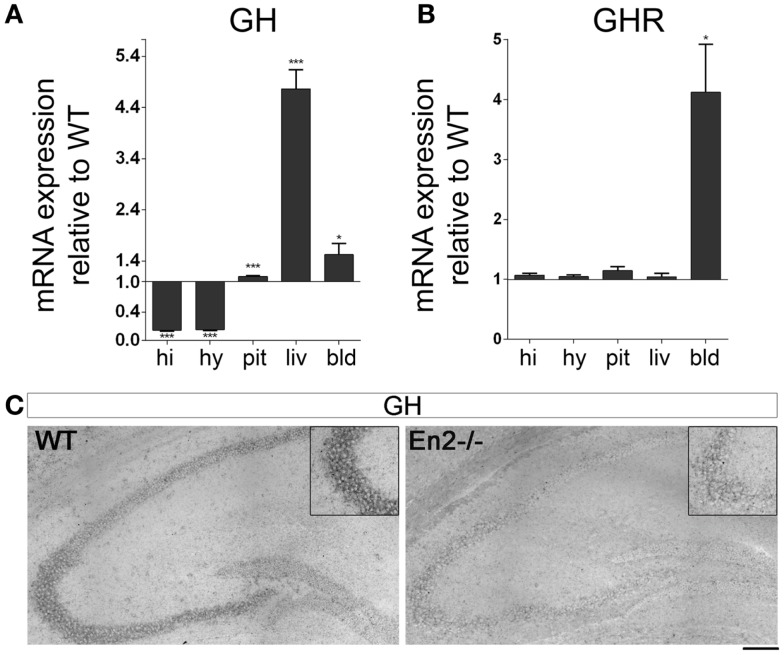
**Expression of GH and GH receptor mRNAs in the neuroendocrine axis of WT and *En2*^−/−^ mice**. **(A)** GH mRNA quantitative RT-PCR. **(B)** GHR quantitative RT-PCR. Values are plotted as each gene/L41 comparative quantitation ratios normalized on the expression of WT (mean ± SEM of three replicates from pools of six animals per genotype; *p < 0.05, ***p < 0.001; Student’s *t*-test, *En2*^−/−^ vs. WT). **(C)** Representative pictures of GH mRNA *in situ* hybridization on the dorsal hippocampus from WT and *En2*^−/−^ mice. Insets show the CA3 subfield. Scale bar: 200 μm (whole hippocampi) and 125 μm (insets). Abbreviations are as in Figure 1.

Before investigating GH, GHR, mGRF, SST, IGF-1, and IGF-1R mRNA expression in the neuroendocrine axis of *En2*^−/−^ mice, we first verified whether *En2* might directly regulate their transcription. Indeed, bioinformatic analysis revealed that *En2* binding sites are present in the promoters of all these genes (Table 2). We next studied GH and GHR mRNA expression in the neuroendocrine axis of WT and *En2*^−/−^ adult mice. We found that GH mRNA expression was markedly deregulated throughout the neuroendocrine axis in *En2*^−/−^ mice. A statistically significant increase of GH mRNA levels was detected in the pituitary gland (+72%, *p* < 0.001), liver (+376%, *p* < 0.001), and blood (+87%, *p* < 0.05) of *En2*^−/−^ mice, as compared to WT littermates (Figure 2A). GH mRNA levels were instead significantly lower in the *En2*^−/−^ hypothalamus (−97%, *p* < 0.001) and hippocampus (−98%, *p* < 0.001), as compared to WT (Figure 2A). *In situ* hybridization confirmed GH mRNA decrease in the *En2*^−/−^ hippocampus, mainly in the CA3 subfield (Figure 2C). No significant differences in GHR mRNA levels were detected between genotypes in the analyzed tissues, with the exception of blood, where a marked increase was detected in mutant mice compared to controls (+312%, *p* < 0.05) (Figure 2B).

**Table 2:** **Presence of *En2* binding sites onto GH, GHR, mGRF, SST, IGF-1, and IGF-1R gene promoters**.

Gene	Accession no.	Start	End	Strand	Core	Matrix	Sequence
GH	GXP_219787	534	552	+	0.752	0.73	agccatgAATAaatgtata
GHR	GXP_889069	584	602	–	1	0.863	ccccataAATTaataatcc
IGF-1	GXP_4345839	966	984	–	1	0.858	ttttatgAATTaagccctc
IGF-1R	GXP_183481	512	530	–	1	0.854	aacattgAATTagttcttg
mGRF	GXP_4357410	266	284	–	1	0.797	ggaaacaAATTgaacaaat
SST	GXP_82140	62	80	–	1	0.774	cagaatgAATTtgcaatta

*The promoter regions of the six genes belonging to the GH/IGF-1 pathway were analyzed for the presence of *En2* binding site using the Matinspector program. Core similarity (Core) indicates the match against the nucleotides representing the core motif of the *En2* binding site (AATT), where 1 is 100% identity. Matrix similarity (Matrix) is the overall similarity to the *En2* binding motif, where 1 is 100% identity. For each sequence, the start/end position respect to ATG is indicated, together with the strand on which they are present. For each promoter, several regions containing the *En2* binding site were found; the table reports only those with highest values for core and matrix similarity. Genomatix Promoter database (GXP) accession numbers are given for each gene. Abbreviations are as in the text*.

### Altered expression of mGRF and SST in the hypothalamus of *En2*^−/−^ mice

**Figure 3:**
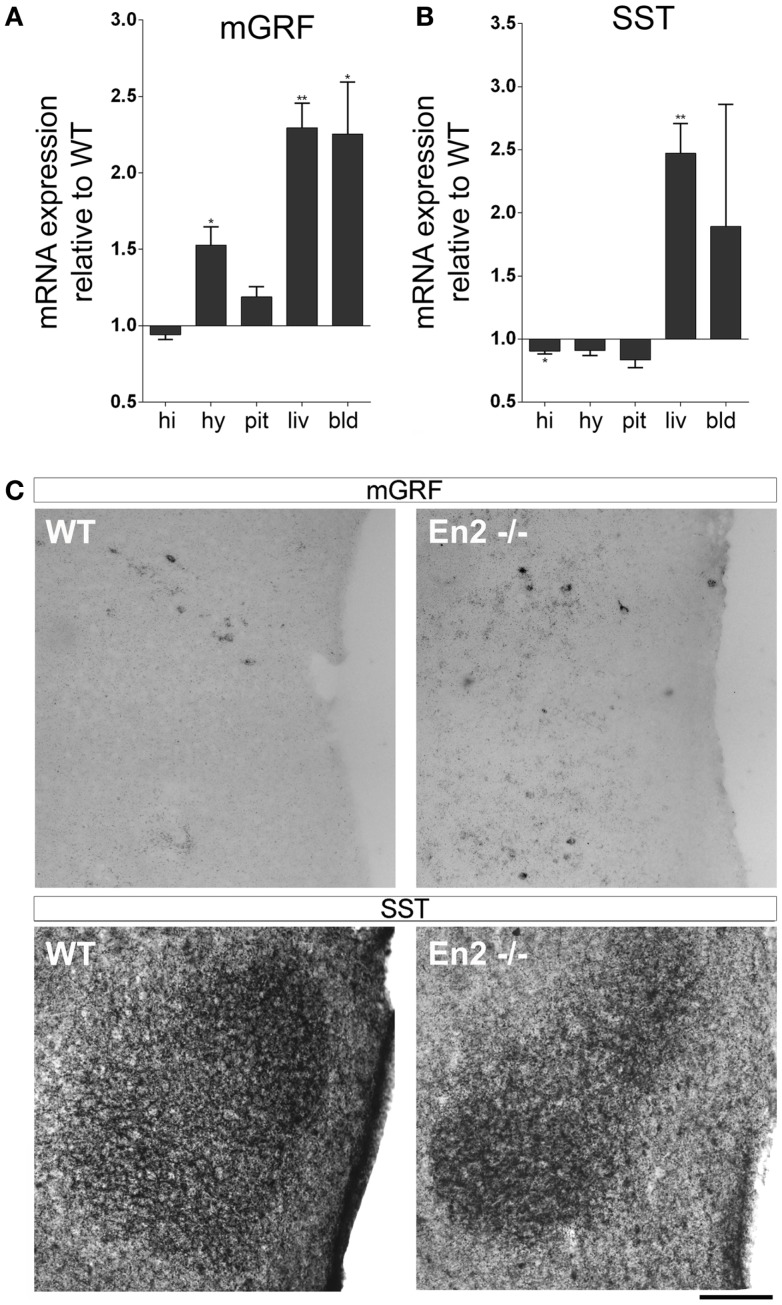
**Expression of mGRF and SST mRNAs in the neuroendocrine axis of WT and *En2*^***−***/−^ mice**. **(A,B)** mRNA expression levels of mGRF **(A)** and SST **(B)**, obtained by quantitative RT-PCR. Values are plotted as each gene/L41 comparative quantitation ratios normalized on the expression of WT (mean ± SEM of three replicates from pools of six animals per genotype; *p < 0.05, **p < 0.01; Student’s *t*-test, *En2*^−/−^ vs. WT). **(C)** mGRF *in situ* hybridization and SST immunohistochemistry. Representative pictures show mGRF mRNA and SST protein staining in the dorsomedial and ventromedial paraventricular nuclei of the hypothalamus. Scale bar: 200 μm. Abbreviations are as in Figure 1.

We then investigated the expression of mGRF (also known as growth hormone releasing hormone, GHRH) and SST, the two hypothalamic hormones regulating GH synthesis, in the brain–pituitary–liver axis of WT and *En2*^−/−^ adult mice. As compared to WT controls, a marked increase of mGRF mRNA (+53%, *p* < 0.05; Figure 3A) and a significant decrease of SST mRNA (−13%, *p* < 0.01; Figure 3B) was found in the *En2*^−/−^ hypothalamus. Significantly higher mRNA levels for the two hormones were also found in blood (mGRF: +125%, *p* < 0.05) and liver (mGRF: +129%, *p* < 0.01; SST: +147%, *p* < 0.001) from *En2*^−/−^ mice (Figures 3A,B). According to our previous study (27), lower levels of SST mRNA were found in the *En2*^−/−^ hippocampus, as compared to WT (−10%, *p* < 0.05; Figure 3B). *In situ* hybridization and immunohistochemistry experiments, respectively, confirmed the increased expression of mGRF mRNA and decreased levels of SST protein in the dorsomedial/ventromedial paraventricular nuclei of the *En2*^−/−^ hypothalamus, as compared to WT (Figure 3C).

### Altered IGF-1 mRNA expression in the brain–pituitary–liver axis of *En2*^−/−^ mice

**Figure 4:**
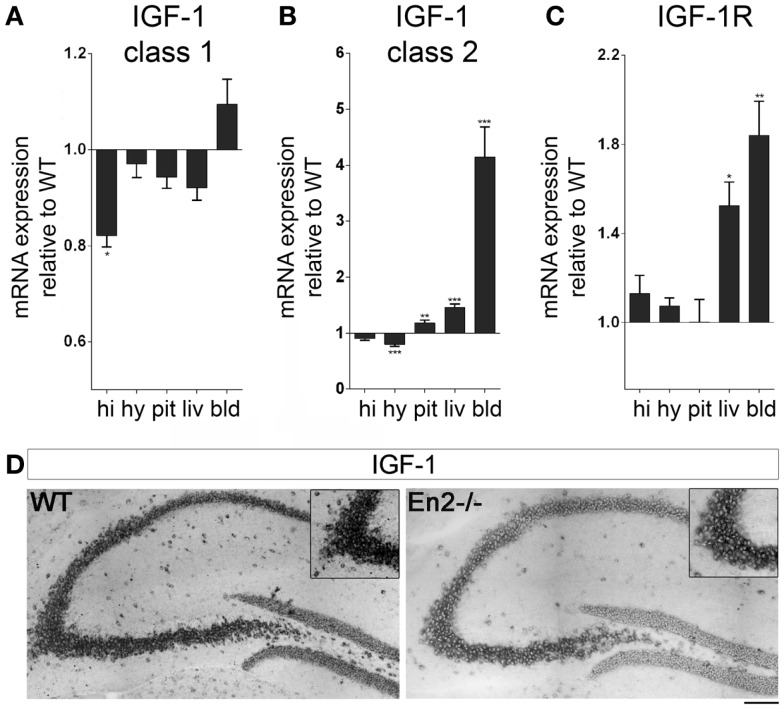
**Expression of IGF-1 and IGF-1R mRNAs in the neuroendocrine axis of WT and *En2*^−/−^ mice**. **(A,B)** Quantitative RT-PCR for IGF-1 class 1 **(A)** and class 2 **(B)** transcripts. **(C)** IGF-1R quantitative RT-PCR. Values are plotted as each gene/L41 comparative quantitation ratios normalized on the expression of WT (mean ± SEM of three replicates from pools of six animals per genotype; *p < 0.05, **p < 0.01; ***p < 0.001; Student’s *t*-test, *En2*^−/−^ vs. WT). **(D)** Representative pictures of *in situ* hybridization for IGF-1 mRNA (both transcripts) on the dorsal hippocampus from WT and *En2*^−/−^ mice. Insets show the CA3 subfield. Scale bar: 200 μm (whole hippocampi) and 125 μm (insets). Abbreviations are as in Figure 1.

We next analyzed mRNA expression of IGF-1 and its receptor in the brain–pituitary–liver axis of WT and *En2*^−/−^ adult mice. IGF-1 mRNA exists in two major forms (class 1 and class 2), class 2 mRNA transcription being directly regulated by GH (33, 34). We found that levels of IGF-1 class 1 mRNA were significantly reduced in the hippocampus but not other tissues of *En2*^−/−^ mice, as compared to WT (−18%, *p* < 0.05 for all comparisons) (Figure 4A). In keeping with GH mRNA expression data (Figure 2), IGF-1 class 2 mRNA was significantly up-regulated in the liver (+43%, *p* < 0.001) and down-regulated in the hypothalamus (−19%, *p* < 0.001) of *En2*^−/−^ mice, as compared to WT littermates; increased levels of IGF-1 class 2 mRNA were also detected in *En2*^−/−^ pituitary gland (+16%, *p* < 0.01) and blood (+312%, *p* < 0.001) (Figure 4B). *In situ* hybridization with a riboprobe specific for both class 1 and class 2 IGF-1 mRNAs confirmed that IGF-1 mRNA levels are decreased in the *En2*^−/−^ hippocampus (Figure 4D). Finally, IGF-1R mRNA levels did not differ between genotypes in hypothalamus, hippocampus, and pituitary gland, while a significant increase was found in blood (+84%, *p* < 0.01) and liver (+52%, *p* < 0.05) from *En2*^−/−^ mice compared to WT controls (Figure 4C). Table 3 summarizes mRNA expression data for all the analyzed genes in the brain–pituitary–liver axis of WT and *En2*^−/−^ adult mice.

**Table 3:** **Summary of the expression of *En2*, GH, GHR, mGRF, SST, IGF-1, and IGF-1R mRNAs in the neuroendocrine axis of *En2*^−/^^−^ mice, as compared to WT**.

Gene	Hippocampus	Hypothalamus	Pituitary	Liver	Blood
GH	−98%***	−97%***	+72%***	+376%***	+87%*
GHR	No difference	No difference	No difference	No difference	+312%*
mGRF	No difference	+53%*	No difference	+129%**	+125%*
SST	−10%*	−13%**	No difference	+147%**	No difference
IGF-1 Class 1	−18%*	No difference	No difference	No difference	No difference
IGF-1 Class 2	No difference	−19%***	+16%**	+43%***	+312%***
IGF-1R	No difference	No difference	No difference	+52%*	+84%**

*Summary of quantitative RT-PCR data. Data are presented as the mean percentage of the decrease/increase of each mRNA in *En2*^−/−^ mice, as compared to WT littermates*.

Statistical significance: *p < 0.05, **p < 0.01, ***p < 0.001 (*En2*^−/−^ vs. WT). Abbreviations are as in the text.

### Reduced levels of GH protein in the *En2*^−/−^ hippocampus

**Figure 5:**
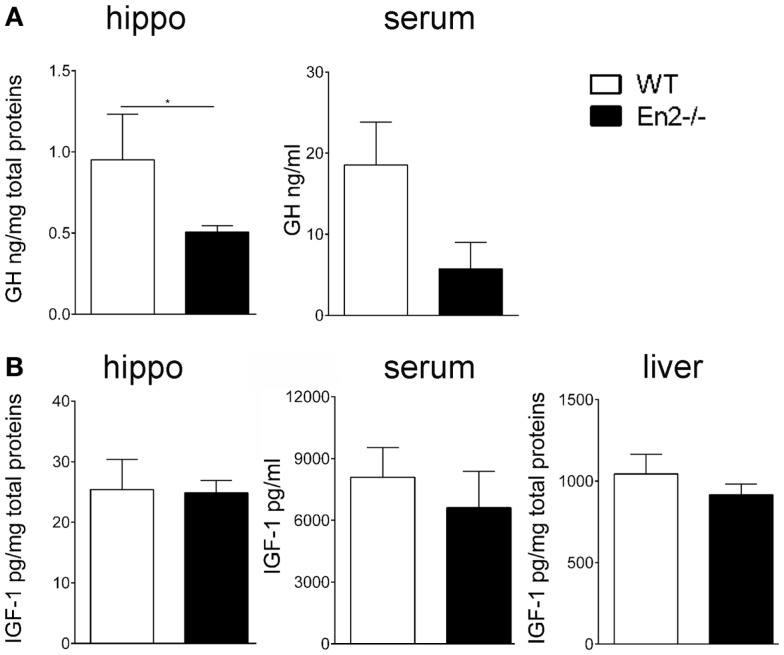
**Levels of GH and IGF-1 hormones in WT and *En2*^−/−^ mice**. **(A,B)** ELISA quantification of GH **(A)** and IGF-1 **(B)** levels in serum and hippocampal (hippo) and liver protein extracts, as indicated. Values are plotted as mean ± SEM (five animals per genotype, in duplicate; *p < 0.05, Student’s *t*-test, *En2*^−/−^ vs. WT). Genotypes are as indicated.

The altered levels of GH and IGF-1 mRNA expression detected in the *En2*^−/−^ neuroendocrine axis prompted us to investigate the levels of these hormones in the hippocampus, serum, and liver of both genotypes. ELISA assays revealed a significant reduction (−54%, *p* < 0.05) of GH protein levels in the hippocampus of *En2*^−/−^ mice, as compared to WT littermates, while no difference was detected in serum samples (Figure 5A). Hippocampal, liver, and serum IGF-1 protein levels did not significantly differ between the two genotypes (Figure 5B).

## Discussion

### Brief summary of results

In this study, we analyzed the expression of GH, IGF-1, their receptors, and regulatory hormones in the brain–pituitary–liver axis of adult *En2*^−/−^ mice, a mouse model for ASD. We found that in mutant mice, GH and IGF-1 mRNA levels were significantly higher in the pituitary gland and liver, respectively, but this increase was not paralleled by higher levels of circulating hormones. In *En2* mutants, GH and IGF-1 mRNA levels were instead significantly down-regulated in the hippocampus, and this reduction was accompanied by a significant decrease of GH but not IGF-1 protein levels.

### *En2* interacts with IGF-1 signaling in the mouse neuroendocrine axis

**Figure 6:**
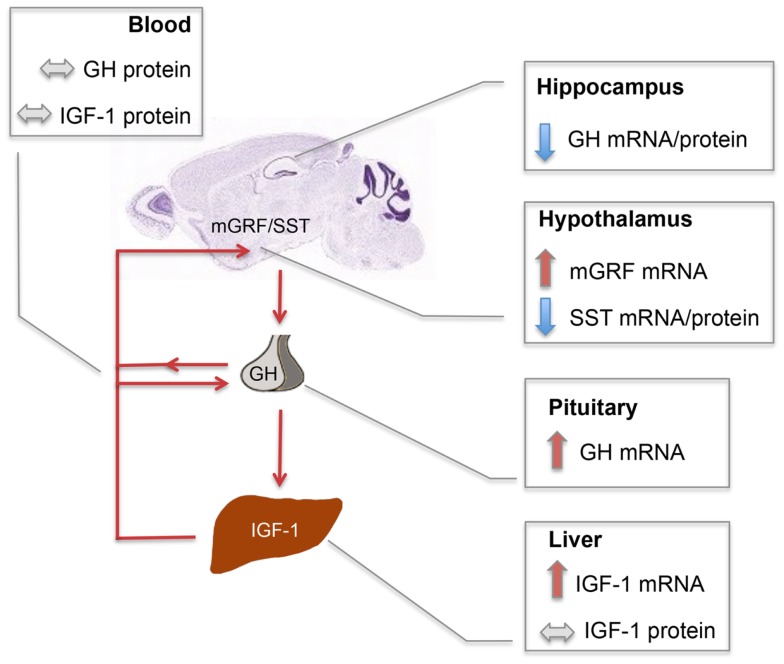
**Schematic summary of SST, mGRF, GH, and IGF-1 expression in the *En2*^−/−^ neuroendocrine axis**. Red and blue arrows, respectively, indicate up- and down-regulations observed in *En2*^−/−^ mice as compared to WT controls. Double-arrowed gray lines indicate comparable levels between WT and *En2*^−/−^ mice. Increased levels of GH and IGF-1 mRNA, respectively observed in *En2*^−/−^ pituitary and liver, are not paralleled by higher levels of circulating hormones, suggesting that a complex post-translational control of GH and IGF-1 synthesis takes place in mutant mice. GH mRNA and protein levels are instead significantly down-regulated in *En2*^−/−^ hippocampus. Arrowed red lines connecting different organs indicate the action of circulating hormones onto their target tissues. The mouse brain sagittal section is a Nissl stain taken from the Allen Mouse Brain Atlas (see text footnote 2). Abbreviations are as in the text.

Our results, schematically summarized in Figure 6, indicate that regulatory mechanisms controlling GH and IGF-1 mRNA expression in the neuroendocrine system are altered in *En2*^−/−^ mice. The presence of *En2* mRNA in the hypothalamus–pituitary–liver axis (Figure 1) and that of an *En2* binding site in the promoters of GH, IGF-1, and other genes of the pathway (Table 2) suggest that *En2* might directly contribute to their transcriptional control. Indeed, recent studies revealed an unprecedented interaction between *En2* and IGF-1 signaling. In the absence of *En2*, IGF-1 has a stronger mitogenic effect on cerebellar GNPs, due the increased activity of downstream effectors of IGF-1 signaling, such as S6 kinase (29). Thus, *En2* appears to negatively regulate IGF-1 signaling during postnatal cerebellar development. Our results strengthen this link between *En2* and IGF-1 signaling, and suggest that a direct transcriptional control of *En2* onto genes belonging to the IGF-1 pathway takes place also in the brain–pituitary–liver axis.

### Differential expression of GH and IGF-1 mRNAs in the brain and peripheral tissues of *En2*^−/−^ mice

IGF-1 mRNA exists in two main isoforms, derived from alternative splicing of the two first exons contained in the 5′ untranslated region (5′ UTR) of the mRNA precursor. Class 1 transcripts contain exon 1, whereas class 2 transcripts contain exon 2 (33). Inclusion of exon 1 or exon 2 is mutually exclusive, resulting in different 5′ UTRs of the IGF-1 mRNA containing different leader sequences of the pre-pro-IGF-I peptide. In addition, transcription of class 1 and class 2 mRNAs is differently controlled; class 2 mRNA being directly regulated by GH. It is, however, important to note that both class 1 and class 2 transcripts code for the same IGF-1 mature peptide (33).

In *En2* mutants, we described a differential expression of IGF-1 mRNAs in different tissues of the brain–pituitary–liver axis. A significant increase of IGF-1 class 2 mRNA was detected in liver and blood, whereas IGF-1 class 1 mRNA was significantly decreased in the *En2*^−/−^ hippocampus (Figure 4). The negative regulation exerted by *En2* onto IGF-1 signaling in the cerebellum (29), therefore, appears to occur also at a transcriptional level in the hippocampus.

Interestingly, *En2*^−/−^ mice showed increased hypothalamic levels of mGRF and decreased levels of somatostatin (SST), the two hormones controlling GH synthesis. Previous studies from our laboratory already showed that loss of *En2* results in lower levels of SST mRNA and protein in the hippocampus and cerebral cortex. Here, we extended this observation, demonstrating that lower levels of SST are also present in the *En2*^−/−^ hypothalamus; hypothalamic levels of mGRF mRNA were instead increased in mutant mice (Figure 3). Deregulation of hypothalamic hormones controlling GH synthesis might result in increased levels of circulating GH and IGF-1 in mutant mice. However, we showed that serum levels of both hormones, as well as IGF-1 levels in liver did not differ between WT and *En2*^−/−^ mice (Figure 5). Indeed, behavioral studies did not reveal gross weight and growth abnormalities in *En2* mutants (23), suggesting that differences in GH and IGF-1 mRNA levels might be blunted at the protein level, via multiple post-transcriptional control mechanisms. Indeed, a tight dependence of IGF-1 translation via several miRNA binding site in the 3′ UTR of the transcripts has been described (33, 35).

### Functional consequences of reduced GH levels in the *En2*^−/−^ hippocampus

The decreased expression of GH mRNA and protein observed in the *En2*^−/−^ hippocampus might contribute to learning deficits observed in *En2* mutants. Indeed, *En2*^−/−^ mice show impaired learning in hippocampal-dependent tasks, such as the Morris water maze and contextual fear conditioning (22, 23). Profound effects on cognitive function have been demonstrated for GH. GH-deficient spontaneous dwarf rats display marked deficits in hippocampus-dependent spatial learning and memory, accompanied by an imbalance in hippocampal glutamatergic/GABAergic synapses and neurogenesis (36). Chronic stress, which is known to affect hippocampal function, also reduces hippocampal GH levels (37). Restoration of normal GH levels in the hippocampus (obtained via viral-mediated gene transfer) is able to reverse stress-dependent behavioral impairment, as tested by hippocampus-dependent tasks (contextual fear conditioning) (37). Taken together, these results indicate that reduced levels of hippocampal GH detected in *En2*^−/−^ mice might contribute to hippocampal dysfunction observed in these mutants.

## Conclusion

To our knowledge, this is the first demonstration that brain GH levels are reduced in a mouse model of ASD. Considering the important role of GH on cognitive functions, our data strengthen the notion that reduced expression of GH in the hippocampus may be implicated in learning disabilities associated to ASD.

## Author Contributions

Giovanni Provenzano and Elena Clementi equally contributed to this study. Giovanni Provenzano designed and performed experiments, analyzed data and wrote the paper. Elena Clementi designed and performed experiments, analyzed data. Sacha Genovesi, Manuela Scali, Prem Prakash Tripathi, and Paola Sgadò performed experiments. Yuri Bozzi provided funding, conceived the study, analyzed data and wrote the paper.

## Conflict of Interest Statement

The Reviewer, Dr Alessandro Sale, declares that despite being affiliated with the same institution as author Dr Manuela Scali and having collaborated with her in the past, the review process was handled objectively and no conflict of interest exists. The authors declare that the research was conducted in the absence of any commercial or financial relationships that could be construed as a potential conflict of interest.

## Footnotes

^1^http://clinicaltrials.gov

^2^http://www.Genomatix.de

^3^http://www.brain-map.org/
